# Dilations and degeneracy in network controllability

**DOI:** 10.1038/s41598-021-88529-5

**Published:** 2021-05-05

**Authors:** Liam Chung, Derek Ruths, Justin Ruths

**Affiliations:** 1 Computer Science, McGill University, Montreal, H3A 0G4 Canada; 2 Mechanical Engineering, University of Texas at Dallas, Richardson, 75080 USA

**Keywords:** Applied mathematics, Computational science

## Abstract

Network controllability asserts a perspective that the structure—the location of edges that connect nodes—of the network contains important information about fundamental characteristics of our ability to change the behavior that evolves on these networks. It can be used, for example, to determine the parts of the system that when influenced by outside controlling signals, can ultimately steer the behavior of the entire network. One of the challenges in utilizing the ideas from network controllability on real systems is that there is typically more than one potential solution (often many) suggested by the topology of the graph that perform equally well. Picking a single candidate from this degenerate solution set over others should be properly motivated, however, to-date our understanding of how these different options are related has been limited. In this work, we operationalize the existing notion of a dilation into a framework that provides clarity on the source of this control degeneracy and further elucidates many of the existing results surrounding degeneracy in the literature.

## Introduction

Since the field of network controllability was stoked back into existence in 2011^1^, there has been a surge in interest to apply techniques from structural control on real systems^2–11^. These efforts have been frustrated by the fact that many degenerate—seemingly equally good—options exist for achieving control over the dynamics of a network. In particular, network controllability enables finding a smallest set of nodes that must be influenced by external inputs in order to steer the state of each node in the network to any arbitrary desired value. The degeneracy of this problem is the fact that there are many different sets of externally controlled nodes that have the same cardinality. Because work to-date has largely taken this *node* perspective, sampling or iteration-based approaches have been used to probe and understand the landscape of control degeneracy^4,9,12^. This perspective effectively works backwards, seeking to summarize and distill insight from the combinatorial explosion of options that compose control degeneracy. These tools are informative and can ultimately produce the degenerate sets of controlled nodes. However, they fail to deliver a comprehensive understanding of the causal source of this degeneracy, especially at a practical level that helps to choose between equivalent control strategies.

In this paper, we advance a framework based on the study of dilations to provide a unifying perspective on degeneracy. Roughly, dilations are the expansion points in the network that create the need for controlling inputs. While the concept of dilations in structural control was made clear at the outset in 1974^13^, it has not been leveraged to identify the origin of equivalent control sets. Here we show that many of the recent observations about network controllability, especially with regard to degeneracy, can be elegantly described through this new framework.

Our dilation-based framework permits the ability to clearly understand which nodes are alternate choices and how these alternate choices are interrelated across the entire network. A dilation-based approach also enables analyzing the controllability of networks under perturbation (e.g., edge removal), by clarifying the role that edges play in granting controllability.

We review the context of the problem, introduce several new definitions and discuss their consequences. Ultimately, we show that this dilation-based framework provides the ability to not only reproduce many of the existing results in the literature but also enhanced insight into the underlying causal mechanisms that give rise to various network control properties.

## Network controllability

The time evolution of a process on a directed network $N=(V,E)$, with vertex set *V* and edge set *E*, can be modeled by a state $x(t) = [x_1(t), x_2(t), \dots , x_n (t)]^T$ that captures the value of the process at each of the *n* nodes at the time step *t*. The update of this state over time can be modeled with linear, time-invariant (LTI) dynamics

1 $$\begin{aligned} x(t+1) = Ax(t), \end{aligned}$$

where the $n\times n$ matrix *A* models the connectivity between nodes (the transpose of the adjacency matrix, $A_{ij}$ is the weight of the edge from node $x_j$ to node $x_i$). While such a LTI model is often a significant simplification of the actual behavior, it allows for analysis of first-order effects, such as the impact of external control on the system. Before more sophisticated models are studied, the properties of linear systems must first be well understood. Here the process evolves freely from an initial condition and the dynamics is characterized completely by the matrix *A*, i.e., $N=(V,E)=G(A)$, where $V=X=\{x_1,\dots ,x_n\}$ and $E=E_A=\{(i,j) : A_{ji}\ne 0\}$. When we seek to control the process behavior, we do so through the application of external inputs $u(t)=[u_1(t),u_2(t),\dots ,u_m(t)]^T$

2 $$\begin{aligned} x(t+1) = Ax(t) + Bu(t), \end{aligned}$$

where the $n\times m$ matrix *B* models the connections between the *m* inputs to the states ($B_{ij}$ is the weight of the edge from the input $u_j$ to the node $x_i$). Now the process is driven by these inputs, which can be considered as a special type of node, which has a node value but is not influenced by other nodes. This interpretation gives rise to an augmented network whose dynamics is characterized by both *A* and *B*, i.e., $N=(V,E)=G(A,B)$, where $V=X\cup U$ and $E=E_A\cup E_B$ with $U=\{u_1,\dots ,u_m\}$ and $E_B=\{(i,j) : B_{ji}\ne 0\}$. We call the selection of input-to-state connections of the control augmented graph a *control configuration*.

One of the most fundamental control properties is controllability, which addresses whether it is possible, through direct manipulation of the input vector *u*(*t*) over some finite period of time, to drive *x*(*t*) to reach any arbitrary vector value in ${\mathbb {R}}^n$. A controllable control configuration is able to change the current network state from any arbitrary initial value $x_0=x(0)$ to any arbitrary final value $x_T=x(T)$ through the application of exogenous inputs *u*(*t*) over the time horizon $t=0,\ldots ,T$. Controllabilty captures the intertwining of the original network *A* and the inputs *B*, so this assessment is for a specific control augmented graph, and for the same network the outcome can change if the control configuration is changed. A control configuration *G*(*A*, *B*) is controllable if and only if the matrix $C = [B, AB, A^2B, \ldots , A^{n-1}B]$, called the *controllability matrix*, is of full rank *n*^14^. The rank operation is sensitive to the structure of *A* and *B* but also to the numeric values of the edge weights. Edge weights are often unknown (or can fluctuate) and for large graphs computing the rank becomes a numerical challenge.

Structural Control is a tool to analyze the control properties of linear time invariant systems using only structural connectivity, the architecture of edges connecting nodes. It assumes structured matrices *A* and *B*, which only captures knowledge of the absence of edges, i.e., fixed zero values of the matrices^13^. This abstraction allows structural control to study systems generically, which studies which topologies have good properties, ignoring pathological cases where edge weight symmetries cause a problem. Structural controllability can be determined by whether the generic rank of the controllability matrix is equal to the number of states, *n*^13,15^. Another distinctive benefit of structural control is that properties can be assessed directly using network algorithms.

Because systems modeled as networks are often composed of actors (nodes) of the same type (e.g., humans in social networks, proteins in biochemical networks) and because these systems were not engineered by humans, the problem of finding a good control configuration often arises in the network context. The control configuration design problem aims to identify the number and the location of external inputs (i.e., choose the matrix *B*) to confer desirable control properties, like controllability. Here, we aim to find the smallest, or minimal, control configuration with the fewest inputs *m* that guarantees (structural) controllability. While the minimum number of controls is a unique integer that is computed from the network structure *G*(*A*), there are typically more than one minimal control configuration that employs the minimum number of inputs^16^. Although this degeneracy provides a great deal of flexibility to the control configuration design problem, it greatly confuses the implementation of controlling a network, since there are many ways to connect inputs to the network. In this work, we provide a framework to not only capture, but also explain, the underlying equivalences between groups of nodes that govern the generation of the degenerate controllable control configurations.

### Dilations

Dilations in networks are defined as sets of nodes that have fewer nodes pointing in to them (in-neighbors) than there are nodes in the set. Intuitively it suggests that since fewer nodes point into the set, there is insufficient information coming into the set to individually distinguish the nodes in the set from one another. In the control context, we look for deficient subsets of the *state* nodes. Given a set of state nodes *S*, we denote *T*(*S*) as the set of in-neighbors, the nodes which have an outgoing edge ending at a node in *S*.

#### Definition 1

In a network $N = (X\cup U,E) = G(A,B)$, we define the in-neighbors of a set of nodes $S \subseteq X$ to be the set $T(S) := \{ v \in V : (v,x) \in E,\ \forall x \in S\}$.

#### Remark 1

It is a direct consequence of the special definition and role of the input nodes *U*, that we do not look for dilations in the input nodes of a control augmented graph. The set $S\subseteq X$ is a set drawn only from the state nodes, however, the in-neighbor set is drawn from both state and input sets, i.e., $T(S) \subseteq V$, $V=X\cup U$.

#### Definition 2

A dilation in a network $N = (X\cup U,E) = G(A,B)$ is a set of nodes $D \subseteq X$ such that $|T(D)| < |D|$.

#### Remark 2

Note that the in-neighbors, *T*(*D*), and the set, *D*, are not necessarily disjoint. A node in *D* with an edge pointing to another node in *D* would be considered an element of *T*(*D*).

**Figure 1:**
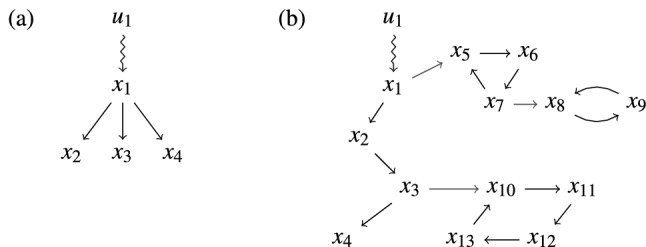
(**a**) Characteristic expansion of a dilation and (**b**) an example of a cactus. The red edges (namely from $x_1$ to $x_5$, $x_3$ to $x_{10}$, and $x_7$ to $x_8$) are the distinguished edges of their respective buds.

Figure 1a provides a schematic for a simple dilation that shows their characteristic expansion. Looking at the “Y” structure, it is intuitive that the in-neighbor $T(D)=\{x_1\}$ to the set $D=\{x_2,x_3,x_4\}$ will not be able to differentiate the states of the nodes in *D*. Although the edge weights from the in-neighbor to the nodes in the set are unknown and may be different, the state values will, for example, all increase or decrease together (assuming all edge weights are the same sign). It is impossible to make the value of node $x_2$ increase and node $x_3$ decrease using only the one in-neighbor (see Supplementary Section S1 for a clear example). Dilations are a fundamental tool for studying controllability from a graphical perspective and is confirmed in Proposition 1 below, which states that (assuming every state node can be reached from at least one input node) a control configuration is controllable if and only if the control configuration has no dilations^13,15^. While this connection between controllability and dilations explains the origin of the need for controls in a network, it is difficult to operationalize for testing controllability or for control configuration design.

### Cacti

If a dilation denotes the inability to control nodes in a network, a cactus captures the portions of the graph that can be controlled. In addition, the concept of a cactus opens the door to graphical algorithms for determining controllability and for facilitating control configuration design. A cactus is a subgraph that is composed of a stem and buds, which we define below.

Recall a path of length $\ell$ is a sequence of nodes $v_1, v_2, \ldots , v_\ell$ such that $(v_i,v_{i+1})\in E$, for $i=1,2,\ldots ,\ell$. A cycle of length $\ell +1$ is a path of nodes $v_1, v_2, \ldots , v_\ell$ with the additional edge $(v_\ell ,v_1)\in E$.

#### Definition 3

Given a network $N = (X\cup U,E_A\cup E_B)$, we define:

1. A stem is a *U*-rooted path—a path whose first node is an input node, $v_1\in U$.
2. A bud is a cycle which has a distinguished edge that connects from one of the following to a node in the cycle: a stem, an input node, another bud.

#### Definition 4

A single cactus subgraph is a stem with any number of connected buds. Multiple vertex-disjoint cactus subgraphs together are called cacti.

#### Remark 3

A cactus is a subgraph of the original graph, which means that there are, in general, additional edges of the graph that are not used to build the cactus, but are still involved in the dynamics of the network.

The definition of stem and cactus (and that multiple cacti are disjoint) indicates that each input node can only be involved in one cactus. Because all nodes must also be reachable by an input node, every bud must be connected (directly or indirectly) to a stem, even if the stem is composed of only an input node. Figure 1b shows an example of a cactus. The following result connects the property of structural controllability to both dilations and cacti.

#### Proposition 1

*For a network and given control configuration* *G*(*A*, *B*), *the following statements are equivalent*:

- *G*(*A*, *B*) *is structurally controllable*.
- *G*(*A*, *B*) *contains no inaccessible nodes and no dilations*^13,15^.
- *G*(*A*, *B*) *is spanned by cacti*^13,17,18^.

While this result provides a way to determine the structural controllability of a given control configuration, it indirectly suggests how to find a control configuration that achieves complete structural controllability. Later work showed that the cacti, based on a collection of disjoint path and cycle families, can be constructed from a maximum (directed) matching of the original graph *G*(*A*)^1,16,19,20^. Recall that a (directed) matching is a set of edges without common vertices at their sources or targets (each vertex can have at most one in and one out edge in the matching), and that a maximum matching is the largest possible matching, given the network structure. Nodes with an inbound edge in the matching are called matched nodes, otherwise unmatched. A control configuration is designed by adding and connecting a new input to each unmatched node, making it a so-called *driver* node. Subsequently, additional edges can be added from the input nodes to cycles to create buds as needed (note that the nodes in a cycle are already matched hence not considered driver nodes). Although the number of edges in a maximum matching is unique for a given network, there is, in general, multiple different sets of edges that may attain a maximum matching. Thus, there are also multiple degenerate cacti and, therefore, multiple control configurations that achieve structural controllability of the network. We call this *control degeneracy*.

If a control configuration *G*(*A*, *B*) is specified, then a weighted maximum matching can be used to ensure that all input nodes are included in the cacti (or, equivalently, to identify which nodes are controllable and which are not)^8,17,19,20^. Matched nodes from a weighted maximum matching are controllable by the given control configuration; unmatched nodes are not. However, there again is degeneracy in the matching and, therefore, degenerate choices in the nodes that can be controlled by the control configuration (although the number of controllable nodes is fixed). In either control configuration design or evaluation of a given control configuration, it is this degeneracy that has caused a large amount of ambiguity in implementing ideas related to controllability of networks. Both sampling-based and enumeration-based approaches have been developed to study the matching directly^21–23^ as well as applying these ideas to network controllability^4,12^. Unfortunately, these approaches do not help to explain the causal structure behind the degeneracy.

If we consider the non-controlled graph *G*(*A*) in Fig. 1a (the subgraph of only state nodes), there are three maximum matchings, each of cardinality one: $M_1=\{(x_1,x_2)\}$, $M_2=\{(x_1,x_3)\}$, $M_3=\{(x_1,x_4)\}$. Since the number of nodes is four and in each matching only one node has an incoming matched edge, three controls are required. The fact that there are three degenerate matchings indicates that there are three different control configurations to confer structural controllability. In the given control configuration selected in Fig. 1a, there are three weighted maximum matchings each of cardinality two: $M_1=\{(u_1,x_1),(x_1,x_2)\}$, $M_2=\{(u_1,x_1),(x_1,x_3)\}$, $M_3=\{(u_1,x_1),(x_1,x_4)\}$. In each degenerate matching, $x_1$ is matched by the incoming edge from $u_1$ and the three matchings selecting $x_2$, $x_3$, and $x_4$ as matched, respectively. This means that $x_1$ is always controllable and that either $x_2$, $x_3$, or $x_4$ can also be controlled by this control configuration, but the number of controllable nodes is always two, which is the number of nodes (4) minus the number of unmatched nodes (2). The difference between controlling $x_2$ versus $x_3$ or $x_4$ is how the actual input sequence *u*(*t*) is designed (see Supplementary Section S2).

A concise and accurate, albeit seemingly non-technical, way to understand control in a network is to consider that each node can “control” only one of its outbound neighbors. Cycles self-regulate, so that a single node can control one outbound neighbor and any number of cycles it has an edge to. One can visualize then, the influence of a control being propagated down the stems of the cacti. When a propagated control reaches a dilation there is a choice as to which node in the dilation it will “choose” to control. The others, for full structural controllability, will need to be controlled by other inputs. While this language of describing the influence of a control omits many of the details mentioned above, it is intuitive and accurate and we will use this throughout the paper with the knowledge that its precise meaning is grounded in the technical details of this section.

## Control degeneracy from the perspective of dilations

We described how the degeneracy caused by multiple equally good maximum matchings creates the possibility of having multiple different minimal control configurations that enable full structural controllability.

### Definition 5

The control degeneracy $\chi _N$ of a network *N* is the number (cardinality of the set) of all possible minimal control configurations of *N*.

Although the connections between dilations, the cacti, and structural controllability are made clear in Proposition 1, to-date the discussion surrounding control degeneracy has been led by observing the degeneracy in the cacti and control configurations by way of the maximum matching. The challenge is that this does not help identify the network structures that lead to this degeneracy. This paper asserts that this degeneracy and much of the work surrounding control degeneracy can be explained elegantly and concisely through the lens of dilations. In order to achieve this aim, we need to select dilations carefully so they are independent and functionally relevant.

### Quantifying the role of a dilation

**Figure 2:**
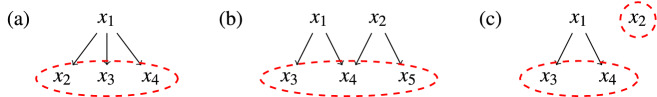
Dilation examples which illustrate the need for updated definitions including Dilation Delta, Minimal Dilation, and Dilation Choice Set. Important dilations are circled sets of nodes.

Dilations identify locations in the network where additional inputs need to be added to make the network structurally controllable. However, not all dilations are equivalent—they vary not only in cardinality of the set *D*, but also the cardinality of the in-neighbor set *T*(*D*). For example, Fig. 2a,b both have dilation sets (circled in red) with $|D|=3$, however, the in-neighbor set $|T(D)|=1$ in Fig. 2a and $|T(D)|=2$ in Fig. 2b. Given our prior discussion, this indicates that these sets require two addition inputs and one additional input, respectively (these are the inputs needed due to the circled dilations; fully controlling these networks would require adding inputs for the other dilations, namely the dilation $\{x_1\}$ in Fig. 2a and $\{x_1\}$ and $\{x_2\}$ in Fig. 2b). We define the dilation delta, or $\Delta _D$, for some dilation *D* to capture this discrepancy between dilation sets.

#### Definition 6

For some dilation *D*, the dilation delta $\Delta _D$ is denoted as $|D| - |T(D)|$.

#### Remark 4

Note that for any set of nodes *S*, $\Delta _S$ can be measured. However, *S* is a dilation if and only if $\Delta _S > 0$.

The utility of the dilation delta is that it represents how many controls will be added to the network for some dilation.

### Identifying minimal dilations

Ideally, we would like the sum of dilation deltas over the whole network to give the minimum number of controls. However, the definition of dilations does not prohibit dilation sets from being combined arbitrarily or overlapping and this could lead to double counting. This motivates us to distill dilations into their most fundamental parts. Consider, for example, Fig. 2c. The sets $\{x_3,x_4\}$ and $\{x_2\}$ are both dilations; however the union $\{x_2,x_3,x_4\}$ is also a dilation. In using dilations as a way to identify locations in the network that require additional inputs, it is misleading to combine these sets because the inputs required to control $\{x_2\}$ are not functionally related to those required by $\{x_3,x_4\}$. To partially alleviate this issue, we can cut a little closer to the core of the network topology with the definition of a minimal dilation (see a similar definition in^24^).

#### Definition 7

A minimal dilation *D* is a dilation such that no proper subset is also a dilation.

#### Theorem 1

*The dilation delta of any minimal dilation* *D* *is* $\Delta _D = 1$.

(Proofs are provided in the Supplementary Section S7).

#### Remark 5

Given a network, the set of minimal dilations is unique.

One of the crucial properties of minimal dilations is that they are modular pieces that can be combined with other dilations (minimal or non-minimal) to yield other dilations. This property is not in general true for any two non-minimal dilations (see Supplementary Section S3).

#### Theorem 2

*Given a network with dilation* *D* *and minimal dilation* ${\tilde{D}}$, *then* $D \cup {\tilde{D}}$ *is a dilation*.

### Identifying independent dilations

Minimal dilations were born out of the idea that some dilations should not be combined. However, there are some that should be. For example, looking again at Fig. 2a, the sets $D_1=\{x_1\}$, $D_2=\{x_2,x_3\}$, $D_3=\{x_3,x_4\}$, and $D_4=\{x_2,x_4\}$ are all minimal dilations. Summing up the dilation deltas yields 4 for the entire network even though only 3 inputs are required to control this network. This discrepancy is caused by the fact that adding inputs to compensate for these dilations must be considered in a particular order and are interrelated. Although selecting inputs for $D_2$, $D_3$, and $D_4$ is contingent on the input selected for $D_1$, there is no ambiguity that an input must be added to $x_1$. Once this input is added, there is degeneracy in where the remaining two inputs should be added. This *choice* between the set $\{x_2,x_3,x_4\}$ is what gives rise for the need to aggregate minimal dilations in a meaningful way.

Having a single common in-neighbor is not the only way to cause this level of interdependency. In Fig. 2b, with more of a “W” structure instead of a “Y” structure, the sets $D_1=\{x_3,x_4\}$ and $D_2=\{x_4,x_5\}$ also lead to similar ambiguity. Notice that once minimal dilations are found, capturing these sets with choices comes down to identifying overlap in the minimal dilations. Thus, we introduce the following definition, and the core concept of this paper: the Dilation Choice Set.

**Figure 3:**
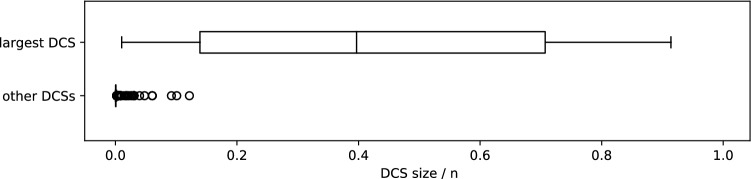
Survey of the dilation choice sets of 17 real networks from various applications and of various sizes. In each network the size of the largest DCS ranged from 1 to 91% of the network size, while the remaining (non-largest) DCSs tended to be small (maximum 12%, average 0.1% of the network size).

#### Definition 8

Denote the minimal dilations of a network as $D_1, D_2, \ldots , D_k$. A single Dilation Choice Set (DCS) is the maximal union of minimal dilations that overlap with at least one other minimal dilation in the union, i.e.,

$$\begin{aligned} D_{i_1} \cup D_{i_2} \cup \dots \cup D_{i_q} \end{aligned}$$

such that for each $i\in \{i_1,i_2,\ldots ,i_q\}$ there exists a $j\in \{i_1,i_2,\ldots ,i_q\}$, $j\ne i$, with $D_i \cap D_j \ne \emptyset$; and *q* is as large as possible. Completing all possible maximal unions over the minimal dilations provides the complete collection of dilation choice sets (see Supplementary Section S4 for a rigorous definition).

#### Remark 6

All dilation choice sets are dilations. This follows from the fact that the union of a dilation with a minimal dilation is a dilation. We refer to any dilation, minimal dilation, or dilation choice set that contains only one node as trivial.

#### Remark 7

By definition, any two dilation choice sets in a network are disjoint.

#### Remark 8

Given a network, the collection of dilation choice sets is unique.

DCSs are built using minimal dilations that overlap, and represent the set of all choices their in-neighbors have for propagating control and choices possible for adding additional inputs to the network. A natural consequence of the definition of the DCS is that all directly controlled nodes, in any minimal control configuration for the network, are in a DCS (since DCSs are composed from dilations).

The DCS captures the degeneracy caused by network structures. Because minimal dilations are small, local structures it may be intuitive to consider DCSs as small, however, Fig. 3 readily demonstrates that the size of the largest DCS in a network can range from small to quite large (see Supplementary Section S5). The majority of the remaining (non-largest) DCSs are typically small. Despite potentially being large, the DCS offers a modular way to select the directly controlled nodes in that the selections made in one DCS can be made independent of the selections made in other DCSs. These choices of directly controlled nodes taken together always lead to the same number of total network controls; different control configurations but all of the same size. Ultimately, it facilitates the discussion of control degeneracy by identifying its fundamental root cause, grounded in the network topology.

## Consequences

In this discussion, we will show ways in which the DCS framework provides an alternate—and possibly more expressive—perspective to understand key attributes of controlling networks. We do this in part by demonstrating how the notion of a DCS can contribute to a unified understanding of the degeneracy by relating and re-explaining as well as extending existing results in the literature.

### Role of nodes and edges in network controllability

When generating a minimum control configuration, it is the maximum matching that dictates the unmatched nodes that receive direct input at the base of the stems of the corresponding cacti (these nodes have often been called “drivers”). Alternative minimum control configurations can be generated by sampling or enumerating alternative maximum matchings^4,5,12^ or by using the notion of the input graph to find alternatives^9^. Because this has largely been an approach based on sifting through the degeneracy, a number of studies have quantified the role that certain nodes and edges play in controlling the network. The DCS concept concisely explains these categories because DCSs are by definition mutually disjoint.

Past work has labeled nodes as critical (*always* a driver), intermittent (*sometimes* a driver), and redundant (*never* a driver)^4^. A DCS framework makes these roles quite clear: a DCS composed of a single node identifies a node that is always a driver; a DCS containing multiple nodes identifies nodes that are sometimes drivers; and nodes not in any DCS are never a driver. Going further, for intermittently controlled nodes, researchers have defined a term “control capacity” as the probability that a node is involved in any given control configuration using a sampling approach^5^. Using our framework, the number of nodes contained within the same DCS can explain the fraction of all minimum control configurations in which an intermittent node participates as a driver (see the discussion on computing degeneracy below for more detail on this).

#### Theorem 3

*A node is intermittent if and only if it is an element of a nontrivial dilation choice set and critical if and only if it is an element of a trivial dilation choice set. Otherwise the node is redundant*.

Edges have similarly been categorized as critical (the number of minimum controls increases after removing an edge), redundant (the same set of minimum controls can be used after removing an edge), or ordinary (all other edges)^1^. This takes a *node-centric* view of categorizing edges by considering how edge removal effects the set of driver nodes. While useful, we show that taking an *edge*-centric view preserves more information and creates more natural division in the roles of edges and their effect on controlling a network. This alternate definition of edge classifications considers the degeneracy directly in terms of the matching and cacti.

In the spirit of the node-centric view of node classifications, edges should be categorized based on the matching degeneracy: edges are critical (*always* in the matching), intermittent (*sometimes* in the matching), or redundant (*never* in the matching). From this perspective critical edges identify parts of the cacti that coincide across all degenerate matchings. Because the maximum matching aims to include as many edges as possible, redundant edges are those that provide “shortcuts” in the graph and are not part of any cacti.

**Figure 4:**
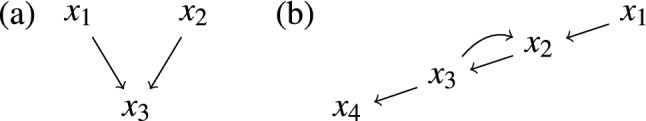
Current categorization of edges takes a node-centric view, however, a new edge categorization from an edge-centric perspective clarifies the roles that edges play in controlling a network.

We use the simple example in Fig. 4a to illustrate the differences in the original node-centric categorization and our new edge-centric categorization. In this example, there is only one minimal control configuration (controlling $x_1$ and $x_2$), but two possible matchings (cacti): either $(x_1,x_3)$ or $(x_2,x_3)$ in which $x_3$ is controlled indirectly via $x_1$ or $x_2$, respectively. Removal of either edge eliminates one of the matchings, however, the control configuration is still structurally controllable. By the current (first) categorization, these edges would be labeled “redundant”, however, they are clearly functional in a different way than the “redundant” edge $(x_3,x_2)$ in Fig. 4b, which is never used in a matching.

To merge both the node- and edge-centric categorizations, we propose a more refined definition in which critical edges appear in the same way, but is more precise in the definitions of redundant and ordinary edges.

#### Definition 9

Consider a network $N=(X,E_A)=G(A)$ with *n* nodes with *m* minimum number of controls required to achieve structural controllability. Let ${\mathcal {M}}_{N,m}$ be the set of all (maximum) matchings of *N* with *m* unmatched nodes and ${\mathcal {B}}_{N,m}$ be the set of corresponding input matrices such that *G*(*A*, *B*) is structurally controllable for all $B\in {\mathcal {B}}_{N,m}$. Suppose we remove an edge $e=(i,j)\in E_A$ and categorize the edge according to the following, letting ${\tilde{N}} = (X,E_A\setminus \{e\}) = G({\tilde{A}})$ be the network *N* with edge *e* removed:

1. An edge *e* is redundant if the following (equivalent) statements hold.
  - Edge *e* is not in any of the degenerate maximum matchings: $e\not \in M,$ for all $M\in {\mathcal {M}}_{N,m}$.
  - The maximum matchings of *N* and ${\tilde{N}}$ are the same: ${\mathcal {M}}_{{\tilde{N}},m} = {\mathcal {M}}_{N,m}$.
  - After removing edge *e*, all original control configurations still achieve structural controllability: ${\mathcal {B}}_{{\tilde{N}},m} = {\mathcal {B}}_{N,m}$.
2. An edge *e* is intermittent if the following (equivalent) statements hold.
  - Edge *e* is in some, but not all of the degenerate maximum matchings: there exist $M_1,M_2\in {\mathcal {M}}_{N,m}$ such that $e\in M_1$ and $e\not \in M_2$.
  - The maximum matchings of ${\tilde{N}}$ are a proper subset of those of *N*: ${\mathcal {M}}_{{\tilde{N}},m}\subsetneq {\mathcal {M}}_{N,m}$.
3. An intermittent edge *e* is matching-disrupting if after removing the intermittent edge *e*, all original control configurations still achieve structural controllability: ${\mathcal {B}}_{{\tilde{N}},m} = {\mathcal {B}}_{N,m}$.
4. An intermittent edge *e* is driver-disrupting if after removing intermittent edge *e*, some, but not all original control configurations still achieve structural controllability: ${\mathcal {B}}_{{\tilde{N}},m} \subsetneq {\mathcal {B}}_{N,m}$.
5. An edge *e* is critical if the following (equivalent) statements hold.
  - Edge *e* is in all of the degenerate maximum matchings: $e\in M,$ for all $M\in {\mathcal {M}}_{N,m}$.
  - There are no maximum matchings of ${\tilde{N}}$ of cardinality *m*: ${\mathcal {M}}_{{\tilde{N}},m}=\emptyset$.
  - After removing edge *e*, there are no structurally controllable control configurations with *m* inputs: ${\mathcal {B}}_{{\tilde{N}},m}=\emptyset$.

In effect, these new definitions have separated the original redundant class of edges into two categories: redundant and matching-disrupting intermittent. The original ordinary edges are now labeled as driver-disrupting. Notice that control degeneracy arises solely from both types of intermittent edges since critical and redundant edges are in all or none of the degenerate matchings, respectively. With this in mind, we see that the DCS framework provides deeper clarity on the role of these edges.

#### Theorem 4

*An edge terminating in a dilation choice set is always intermittent*.

The simple example in Fig. 4a already demonstrated that (matching–disrupting) intermittent edges do not necessarily terminate in a DCS. The definition of driver-disrupting intermittent edges, however, allows us to make this more precise (Supplementary Section S6 provides an example of why the following result is not an exact condition).

#### Theorem 5

*A driver-disrupting intermittent edge always terminates in a dilation choice set*.

These two theorems clearly show that all critical and redundant edges exist outside of DCSs, in the deterministic parts of the network, meaning intermittent edges capture all of the degeneracy. Moreover, because of the tight connection between intermittent edges and the DCS, we can now visualize control degeneracy as relatively local regions of degeneracy interconnected by connectivity that does not change across the matchings and cacti. We introduce the distinction between matching- and driver-disrupting intermittent edges to distinguish between degeneracy of the cacti and degeneracy of the minimal control configuration. These edge classifications and their locations in the network relative to the DCSs help to provide substantiation for the in- and out-degree correlations observed with the number of controls required for structural controllability^3^.

### Global degeneracy from DCS degeneracy

Nodes within a DCS are alternative choices for the drivers of network control. These choices across all DCSs combine to reveal the true source of the degeneracy that appears in the maximum matching. DCSs decompose the network into sets of control-related nodes which can be used to determine how they contribute to the overall degeneracy due to their mutually disjoint nature.

Global degeneracy is the degeneracy $\chi$ of the whole network. The minimality and disjointness properties of DCSs allows for the calculation of global degeneracy to be straightforward. It is the product of the DCS degeneracy of every DCS in the network. We then conclude that $\chi = \chi _{D_1} \chi _{D_2} \cdots \chi _{D_k}$, where $D_1, D_2, \ldots , D_k$ are the DCSs of the network, and $\chi _{D_1}, \chi _{D_2}, \ldots , \chi _{D_k}$ are their local degeneracies.

The DCS degeneracy is the degeneracy $\chi _{D_i}$ of just one DCS within the context of a larger network. In the simple case where a DCS has one in-neighbor the DCS degeneracy is the number of nodes in the DCS, because the in-neighbor can control one node in the DCS and the rest must be directly controlled. However, when there are more in-neighbors it becomes more intricate. If the all in-neighbors (say $\rho$ in-neighbors) pointed to all nodes in the DCS (say *r* nodes in the DCS), then the DCS degeneracy can be computed as $$\left( {\begin{array}{c}\rho \\ r\end{array}}\right)$$. However, in the more general case not all in-neighbors can reach all nodes in the DCS and $$\left( {\begin{array}{c}\rho \\ r\end{array}}\right)$$ serves as an upper bound on the DCS degeneracy. The more general case can be handled through a recursive descent through the in-neighbors and we provide a simple algorithm in the Supplementary Information (Supplementary Section S7) to illustrate how this calculation could proceed.

As discussed earlier, control capacity was introduced as the “probability a node will be a driver”, under the assumption that all degenerate control configurations are equally likely^5^. A similar notion of control backbone was also used to quantify the role of nodes as drivers in the context of degeneracy^7^. The calculation of a node’s probability to be a driver is effectively a calculation of the fraction of all minimal control configurations in which the node is a driver. Past work has calculated this number through a sampling approach, however, the independence and disjoint nature of DCSs make this calculation quite simple. A result is that the control capacity, using DCSs, enables it to be a modular computation. To make this concrete, let *v* be a node in a DCS $D_i$, $\chi _v$ be the number of minimal control configurations which contain *v* as a driver, and $\chi _{D_i,v}$ be the number of ways *v* can be selected as a driver within $D_i$. Then the control capacity of node *v* is

$$\begin{aligned} c_v = \frac{\chi _v}{\chi } = \frac{\chi _{D_1} \chi _{D_2} \ldots \chi _{D_{i-1}} \chi _{D_i,v} \chi _{D_{i+1}} \ldots \chi _{D_k}}{\chi _{D_1} \chi _{D_2} \ldots \chi _{D_k}} = \frac{\chi _{D_i,v}}{\chi _{D_i}}, \end{aligned}$$

which reduces the global definition on $\chi _v$ and $\chi$ to a calculation on $\chi _{D_i,v}$ and $\chi _{D_i}$, both of which can be calculated from the discussion above.

### Deficient (uncontrollable) control configurations and robustness

A control configuration is *not* fully controllable if there are too few inputs connected to the network (or connected in poorly chosen ways). Graphically this can either be due to nodes not being reachable from the inputs at all (e.g., a completely disconnected node or separate component) or multiple nodes being driven by too few inputs (e.g., the classic “Y” dilation in Fig. 2). In this latter case, the node values will always be related to each other, and thus the network cannot be fully controllable (see Supplementary Section S1). Past work aimed to quantify the nodes that are controllable by a given node or node set using the notion of control range^25^ and structural reachability by way of a weighted maximum matching algorithm^8,26^. The DCS framework, again leveraging their independent and disjoint nature, clarifies the scope of dependent state values in uncontrollable control configurations. If a node is an element of a DCS, its state value is algebraically related to that of any other uncontrolled nodes in the DCS (if it is the only node in a DCS, then it receives no control).

One of the ways in which a network can become uncontrollable is through changes in the graph. Adding edges never causes issues for structural controllability, but edge removal can. A number of studies have aimed to identify the properties of edges that, when removed, cause control configurations to become uncontrollable or require an increase in the number of control inputs, thus quantifying the robustness of the network control configuration. Such attempts have included categorizing edges based on their effect on the number of controls after removal^1^; quantifying the increase in number of controls required to maintain controllability as edges are removed in various random and targeted ways^27–29^; and quantifying the decrease in reachability of a network under edge percolation^8,26^. As already alluded to before, the DCS-motivated edge classification we presented earlier helps to clearly elucidate the roles of edges based on their impact to not only the control configurations, but also the matching.

### Input graph

The *input graph* of a directed network is defined as an undirected graph with the same nodes and whose edges encode alternative driver nodes that make the directed network structurally controllable^9^. It is built by connecting two nodes $x_i$ and $x_j$ in the input graph if there exists a node $x_k$ which has an edge (in the original network) to both $x_i$ and $x_j$, where one node is matched and the other is not. Doing so requires effectively enumerating through the alternative matchings. The utility of this construction is that it identifies interchangeable driver nodes and with an extension identifies nodes that are never selected as driver nodes across all possible maximum matchings.

The difficulty in using the input graph for deeper understanding of network controllability, is that it is distilled from the degeneracy of the matching rather than built from the source of the degeneracy: dilations. This is underscored in the description of the input graph as capturing “correlation of [minimum input sets] and nodes in control”^9^; whereas here the DCS offers a causal explanation for the nodes involved in network control. Therefore, despite some very direct parallels between the dilation choice sets and the input graph (in fact DCSs can be found from the input graph), a DCS perspective offers additional insight. Because the input graph was devised from the top-down instead of from the bottom-up, it stops short in making the same connections we are able to extract from a dilation-based approach. Most notably, the input graph omits the topology of the dilation in how in-neighbors of the dilation are connected to the nodes in the dilation. This topology is crucial in understanding, for example, the prevalence of how often a node appears as a driver. Ultimately, the input graph is a node-centric point of view, while as we showed earlier, the DCS is an edge-centric point of view.

## Conclusion

In this paper, we have described a view of structural controllability of networks that focuses on dilations, specifically addressing the theoretical and practical challenge of control degeneracy. By beginning with dilations, we provide a framework that explains many of the existing observations made in the literature and also motivates several new definitions and observations. Critically, because we start at the source of the degeneracy, our definition of dilation choice sets offers a modular and disjoint way of constructing and understanding control degeneracy. We show that the combinatorial explosion of options we deal with is actually the interaction of fewer independent and spatially distinct centers of alternatives.

## Supplementary information

Supplementary Information.
