# Supplementary material for: Dilations and degeneracy in network controllability
Source: Sci Rep. 2021 May 5;11:9568. doi: 10.1038/s41598-021-88529-5 (PMC8100115; doi:10.1038/s41598-021-88529-5)
Supplement: Supplementary file 1 — Supplementary Information. [file 41598_2021_88529_MOESM1_ESM.pdf]

Supplementary Information

Dilations and Degeneracy in Network Controllability

S1 Example of Algebraic Dependency in Dilation Control

Here we demonstrate a simple example of how a dilation without a sufficient number of inputs leads to algebraic dependencies in the values of the state nodes. Consider the small network below.

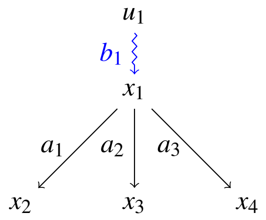

In this simplified example, we can state the value of each node in terms of our other nodes,

$$x_1(t+1) = b_1 \cdot u_1(t),$$
$$x_2(t+1) = a_2 \cdot x_1(t),$$
$$x_3(t+1) = a_3 \cdot x_1(t),$$
$$x_4(t+1) = a_4 \cdot x_1(t).$$

From here it is easy to observe that in every time step, there is a strict algebraic relationship between all three nodes in the dilation,

$$\frac{1}{a_2} x_2(t) = \frac{1}{a_3} \cdot x_3(t) = \frac{1}{a_4} \cdot x_4(t)$$

From this we can easily devise any number of examples that show this system is not controllable. If, for example, all $a_1, a_2, a_3 > 0$ are positive and if the value of x_1 increased then at the next time step all three node states would also increase in value. It would be impossible, then, to increase the value of x_2 but decrease the value of x_3 and x_4. Thus because all final states are not possible to reach, the network with this control configuation is not fully controllable.

S2 Effect of Control Configuration Choice on the Exact Sequence of Inputs

Here, we will demonstrate the practical difference there is between different degenerate control configurations. Here we again use the example given above (taking $b_1 = 1$ for simplicity). It is sufficient to consider that all nodes have zero initial state value, $x(0) = [0,0,0,0]^T$. For some finite t we need to design inputs $u(0), u(1), \ldots, u(t-1)$ such that, $x(t) = [\alpha_1, \alpha_2, \alpha_3, \alpha_4]^T$. As discussed in the paper, there are three minimal control configurations (with all input-to-state edges taken to be weight 1 for convenience):

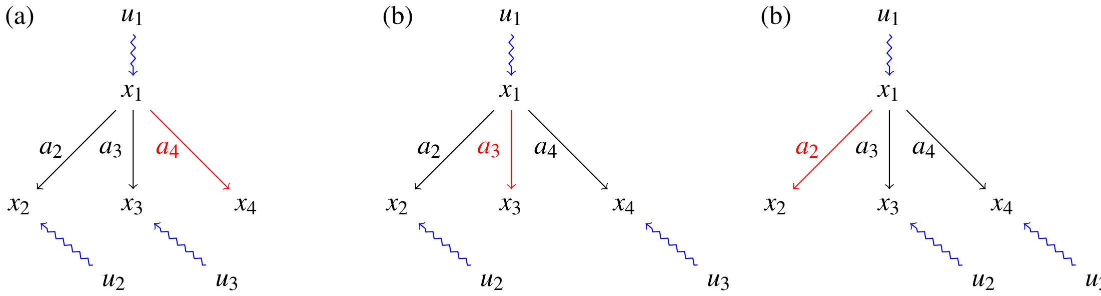

In each configuration, we will directly control two of the nodes in the dilation and the last will be controled via x_1. We will examine configuration (a) first. Our desired value for x_2 and x_3 are α_2 and α_3, and these will be easy to attain, since we directly

control them and they have no out edges, so they're not influencing any other nodes. Thus at the final time step we will set u_2 and u_3 as α_2 and α_3 respectively, but also account for their influence by x_1 by subtracting the designed value for x_1, also accounting for the weights a_2 and a_3. Then for x_4, where our target value is α_4, we need to influence it with x_1. Thus at the first time step we'll have u_1 be $\frac{\alpha_4}{a_4}$. Then at the next time step, we'll let it be α_1, and we're done. Taking this into account for x_2 and x_3, we set u_2 and u_3 to $\alpha_2 - a_2\alpha_4$ and $\alpha_3 - a_4\alpha_4$ respectively.

This intuitive approach can also be captured mathematically by the states at $t = 1$

$$x_1(1) = u_1(0),$$
$$x_2(1) = u_2(0) + a_2x_1(0) = u_2(0),$$
$$x_3(1) = u_3(0) + a_3x_1(0) = u_3(0),$$
$$x_4(1) = a_4x_1(0) = 0,$$

and the states at $t = 2$

$$x_1(2) = u_1(1),$$
$$x_2(2) = u_2(1) + a_2x_1(1) = u_2(1) + a_2u_1(0),$$
$$x_3(2) = u_3(1) + a_3x_1(1) = u_3(1) + a_3u_1(0),$$
$$x_4(2) = a_4x_1(1) = a_4u_1(0).$$

If we select $t = 2$ as the terminal time (so $x_i(2) = \alpha_i$ for $i = 1,2,3,4$), we have four equations with four unknowns $u_1(0)$, $u_1(1)$, $u_2(1)$, and $u_3(1)$, which can be rewritten in matrix form as

$$\begin{bmatrix} x_1(2) \\ x_2(2) \\ x_3(2) \\ x_4(2) \end{bmatrix} = \begin{bmatrix} \alpha_1 \\ \alpha_2 \\ \alpha_3 \\ \alpha_4 \end{bmatrix} = \begin{bmatrix} 0 & 1 & 0 & 0 \\ a_2 & 0 & 1 & 0 \\ a_3 & 0 & 0 & 1 \\ a_4 & 0 & 0 & 0 \end{bmatrix} \begin{bmatrix} u_1(0) \\ u_1(1) \\ u_2(1) \\ u_3(1) \end{bmatrix}. \tag{1}$$

This system of equations can then be solved to produce the input sequences discussed above. A similar analysis is possible for control configurations (b) and (c), and results in the following sequences of inputs.

t	u_1	u_2	u_3
1	$\frac{\alpha_4}{a_4}$	$*$	$*$
2	α_1	$\alpha_2 - a_2\alpha_4$	$\alpha_3 - a_4\alpha_4$

t	u_1	u_2	u_3
1	$\frac{\alpha_3}{a_3}$	$*$	$*$
2	α_1	$\alpha_2 - a_3\alpha_3$	$\alpha_4 - a_3\alpha_3$

t	u_1	u_2	u_3
1	$\frac{\alpha_2}{a_2}$	$*$	$*$
2	α_1	$\alpha_3 - a_2\alpha_2$	$\alpha_4 - a_2\alpha_2$

S3 Combining of Non-Minimal Dilations

The following diagram is an example illustrating that the union properties of minimal dilations (Theorem 2) do not necessarily hold if the dilations are not minimal. Both sets of nodes circled in red are dilations (both being three nodes, with two in-neighbors), but are not minimal (x_6 and x_7 are dilations on their own) and their union circled in black is not a dilation (four nodes with four in-neighbors).

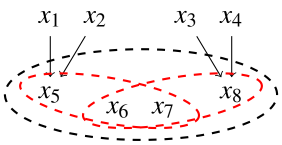

S4 Dilation Choice Sets from a Hypergraph

A hypergraph permits a crisp definition the dilation choice sets of a network. Given the minimal dilations of an original directed network, $D_1, D_2, \ldots, D_k$, we define the set of nodes for the hypergraph H as $\mathscr{V} = D_1 \cup D_2 \cup \cdots \cup D_k$ as the collection of original network nodes that are in any of the minimal dilations. We then create hyperedges (edges that can connect more than two nodes) in the hypergraph to be defined by the minimal dilations. Dilation choice sets are then the connected components of the hypergraph H. Note that trivial dilations (minimal dilations containing a single node) will be isolated nodes in the hypergraph and for the purposes of this analysis will also define a trivial dilation choice set.

S5 Empirical Results

new section

Our empirical results are supported by a non-optimized approach to find the dilation choice sets of a network. This approach leverages the sampling method presented by Jia et al to approximate the control capacity of a node. Although we use this sampling approach, we have a crisp stopping criteria which will allow us to know with full certainty whether we have found all potential control nodes through the sampling approach. Once a DCS has been identified, it is simple to compute the dilation delta as the difference between the cardinality of the DCS $|D|$ and the inbound neighbor set $|T(D)|$. If all DCSs have been found correctly the sum of these dilation deltas will equal the minimum number of controls required to enable network controllability.

// Graph: G
function find_dcss(G)
$\mathscr{D} \leftarrow$ enumerate all possible controls using sampling method in *Jia et al (2013)*[1]
$G' \leftarrow$ subgraph of G induced by $\mathscr{D} \cup T(\mathscr{D})$
$\mathscr{C} \leftarrow$ weakly connected components of G'
dcss $\leftarrow \{\}$
for $c \in \mathscr{C}$ **do**
| dcss.add($c \cap \mathscr{D}$)
end
return dcss

Algorithm 1: Calculating dilation choice sets of a network.

With this algorithm, we compute the dilation choice sets for the following seventeen real networks:

Airport Networks: `intl`, `us`[2] This collection provides two graphs of airport networks. In both, vertices represent airports, and edges exist wherever there were flight(s) between the airports. The first graph represents the complete US airport network in 2010. The second is a graph of international airports. These are available online: http://toreopsahl.com/datasets/.

C. Elegans: `celegans`[3] This collection contains a graph of Caenorhabtitis elegans (C. elegans) worm's neural network. Neurons are vertices and edges indicate existance of at least one synapse or gap junction between neurons. This network is available online: http://toreopsahl.com/datasets.

Circuit Networks: `s208`, `s420`, `s838`[4] Three network representations of electronic circuits parsed from the ISCAS89 benchmark collection (S8). They are available online: http://www.weizmann.ac.il/mcb/UriAlon/.

Corporate Ownership: `eva`[5] This collection consists of ownership relations among companies, where a directed link indicates that the source is an owner of the target. This network is available online: http://vlado.fmf.uni-lj.si/pub/networks/data/econ/Eva/Eva.htm.

E-coli: `ecoli`[6] This collection contains a transcriptional regulation network for E. coli encoding 577 interactions between 116 transcription factors and 419 operons. It represents significant augmentation on top of the existing RegulonDB database. This network is available online: http://www.weizmann.ac.il/mcb/UriAlon/.

Email-EU: `email`[7] This collection includes the network generated from email data from a large European research institution over a period from October 2003 to May 2005. This network is available online: http://snap.stanford.edu/data/.

Food Web: `florida`[8–12] This collection is a collection of various food web networks mostly from marine ecosystems. The orientation of this network is such that directed edges point towards the flow of biomass, e.g., edges point from prey to predators. They are part of the Pejak data collections and are available online: http://vlado.fmf.uni-lj.si/pub/networks/data/bio/foodweb/foodweb.htm.

Gnutella Networks: `p2p-Gnutella04`[7,13] This collection includes a sequence of snapshots of the Gnutella peer-to-peer file sharing network from August 2002. There are total of 9 snapshots of Gnutella network collected in August 2002. Nodes represent hosts in the Gnutella network topology and edges represent connections between the Gnutella hosts. These are available online: http://snap.stanford.edu/data/.

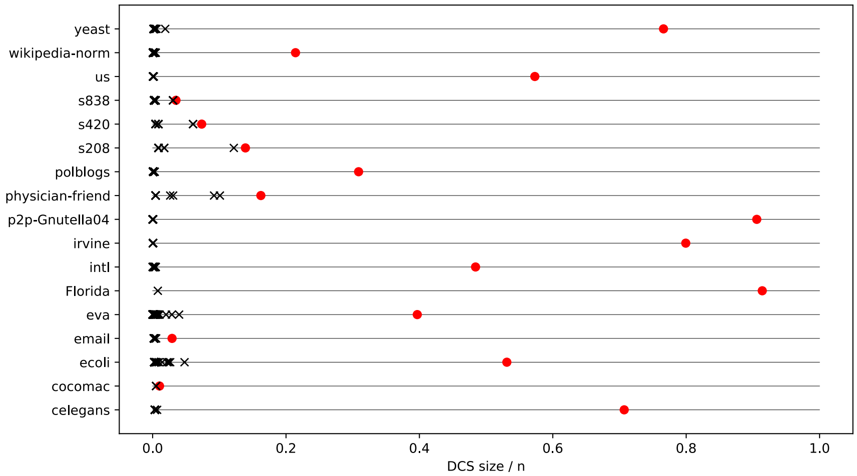

Figure 1. Survey of the dilation choice sets of 17 real networks from various applications and of various sizes. In each network the size of the largest DCS (red •) ranged from 1% to 91% of the network size, while the remaining (non-largest) DCSs (black ×) tended to be small (maximum 12%, average 0.1% of the network size).

Macaque Neural: `cocomac`[14] This collection contains three networks from separate sources, all representing structural (axon projections) cortical connectivity in Macaque monkeys. This networks are available online: `http://cocomac.g-node.org/`.

Physician:[15] We use the `physician-friend` network from this collection, which includes three networks relating to the interactions of physicians in four towns in Illinois, namely who they sought advice from, who they shared cases with, and who are their friends. The edges in these networks have been reversed in order to capture the true direction of influence. This network is available online: `http://moreno.ss.uci.edu/data.html`.

Political Blog: `polblogs`[16] This datset provides a directed graph of links between blogs on US politics in 2005. The edges in this network have been reversed in order to capture the true direction of influence. This network is available online: `http://www-personal.umich.edu/~mejn/netdata/`.

UC Irvine: `irvine`[17] This collection represents the messaging patterns between users of a Facebook-like social network at University of California, Irvine. This network is available online: `http://toreopsahl.com/datasets/`.

Wikipedia-Norm: `wikipedia-norm`[18] The hyperlink network for 1976 nodes describing the social norms of Wikipedia. This network is available online: `http://tuvalu.santafe.edu/~simon/styled-9/styled-10/`

Yeast: `yeast`[19] This collection describes the directed interactions in the yeast transcription network. This network is available online: `http://www.weizmann.ac.il/mcb/UriAlon/`.

Figure 1 shows the network-by-network breakdown of DCS sizes.

S6 Intermittent Edges

Here we present a simple counter example to justify why edges terminating in a dilation choice set can be matching- or driver-disrupting intermittent edges. To demonstrate this, we can consider the following simple example.

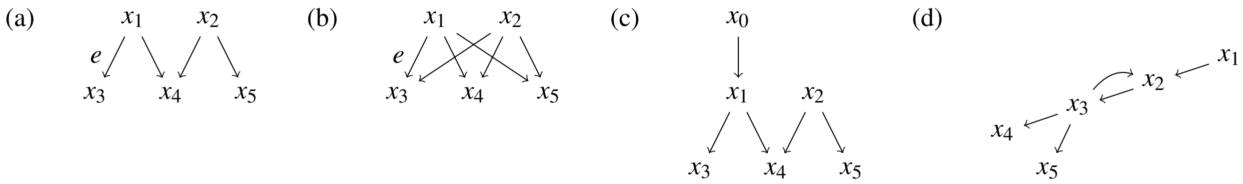

In both (a) and (b) the nodes x_1 and x_2 are critical nodes, being part of trivial dilation choice sets, hence must be drivers. The non-trivial DCS $D = \{x_3, x_4, x_5\}$ has dilation delta $\Delta_D = 1$, which indicates that 1 node must be a driver from D. Hence the potential minimum control configurations are:

- $\{x_1, x_2, x_3\}$,
- $\{x_1, x_2, x_4\}$,
- $\{x_1, x_2, x_5\}$.

The edge we will consider is edge $e = (x_1, x_3)$, which terminates at the DCS, $x_3 \in D$.

In (a), the minimum control configurations correspond to certain matchings:

- $\{x_1, x_2, x_3\}$ corresponding to the matching $M_1 = \{(x_1, x_4), (x_2, x_5)\}$,
- $\{x_1, x_2, x_4\}$ corresponding to the matching $M_2 = \{(x_1, x_3), (x_2, x_5)\}$,
- $\{x_1, x_2, x_5\}$ corresponding to the matching $M_3 = \{(x_1, x_3), (x_2, x_4)\}$.

We call the collection of all matchings $\mathscr{M} = \{M_1, M_2, M_3\}$. If e is removed in (a), then this eliminates two matchings and invalidates two of the minimum control configurations leaving only

- $\{x_1, x_2, x_3\}$ corresponding to the matching $M_1 = \{(x_1, x_4), (x_2, x_5)\}$,

because x_3 must now be a driver. Hence a minimum control configuration with the same number of drivers $m = 3$ can be found, however, the control degeneracy — the number of possible minimum control configurations — has reduced.

In (b), the minimum control configurations correspond to different matchings:

- $\{x_1, x_2, x_3\}$ corresponding to the matching $M_{11} = \{(x_1, x_4), (x_2, x_5)\}$ or $M_{12} = \{(x_2, x_4), (x_1, x_5)\}$,
- $\{x_1, x_2, x_4\}$ corresponding to the matching $M_{21} = \{(x_1, x_3), (x_2, x_5)\}$ or $M_{22} = \{(x_2, x_3), (x_1, x_5)\}$,
- $\{x_1, x_2, x_5\}$ corresponding to the matching $M_{31} = \{(x_1, x_3), (x_2, x_4)\}$ or $M_{32} = \{(x_2, x_3), (x_1, x_4)\}$.

If e is removed in (b), then this eliminates two matchings and does not invalidate any minimum control configurations leaving

- $\{x_1, x_2, x_3\}$ corresponding to the matching $M_{11} = \{(x_1, x_4), (x_2, x_5)\}$ or $M_{12} = \{(x_2, x_4), (x_1, x_5)\}$,
- $\{x_1, x_2, x_4\}$ corresponding to the matching $M_{22} = \{(x_2, x_3), (x_1, x_5)\}$,
- $\{x_1, x_2, x_5\}$ corresponding to the matching $M_{32} = \{(x_2, x_3), (x_1, x_4)\}$.

Hence any of the original minimum control configurations are still valid, hence the control degeneracy — the number of possible minimum control configurations — has stayed the same.

Note that in both cases the number of matchings reduced (this is part of what makes it an intermittent edge in both cases), however, in (a) edge e is driver-disrupting intermittent because it changes the control degeneracy and in (b) edge e is matching-disrupting intermittent because it does not change the control degeneracy.

Finally, we look at two somewhat similar simple examples to contrast the role of intermittent edges with critical and redundant edges. In (c) we observe that edge (x_0, x_1) is always in the matching and thus removing it eliminate all matchings and invalidate all minimum control configurations, thus requiring the number of driver nodes to increase; and in (d) the edge (x_3, x_2) is never in the matching and thus removing it does nothing to the existing maximum matchings or minimum control configurations.

S7 Algorithm for DCS Degeneracy

The following algorithm is a simple approach for calculating the DCS degeneracy of a dilation choice set, i.e., the number of ways that controls can be selected to grant structural controllability. When there is only one in-neighbor of the DCS or if all in-neighbors point to all nodes in the DCS the calculation is simple. When in-neighbors point to different subsets of nodes in the DCS the following algorithm can be used to count all control configurations without double counting.

```
// Graph: G
// in-neighbors of U: S = {s_1, s_2, ..., s_n}
// targets of U: T = {t_1, t_2, ..., t_m}
function calc_degen(G,S,T)
total ← 0
for s ∈ S do
    for t ∈ T do
        if (s,t) ∈ G then
            total ← total + calc_degen(G, S \ {s}, T \ {t})
        else
            continue
        end
    end
end
return total
```

Algorithm 2: Calculating DCS degeneracy of a dilation choice set, U.

S8 Proofs and Extra Results

Theorem 1 *The dilation delta of any minimal dilation D is $\Delta_D = 1$.*

Proof: Let D be some minimal dilation, so by definition the subset $D \setminus \{d\}$ is not a dilation, for any $d \in D$. Since it is not a dilation,

$$|D \setminus \{d\}| \leq |T(D \setminus \{d\})|. \tag{2}$$

Note that since $D \setminus \{d\} \subseteq D$ then the in-neighbor set is also a subset $T(D \setminus \{d\}) \subseteq T(D)$ and, therefore, should have a cardinality no bigger than the original in-neighbor set,

$$|T(D \setminus \{d\})| \leq |T(D)|. \tag{3}$$

Stringing these observations together, we have

$$\begin{aligned} |T(D) &< |D| && D \text{ is a dilation} \\ |T(D)| - 1 &< |D| - 1 \\ &= |D \setminus \{d\}| \\ &\leq |T(D \setminus \{d\})| && \text{by (2)} \\ &\leq |T(D)| && \text{by (3)} \end{aligned} \tag{4}$$

Thus we have that $|T(D)| - 1 < |D| - 1 \leq |T(D)|$. This implies that $|D| - 1 = |T(D)|$, in other words that $\Delta_D = 1$. ■

Theorem 2 *Given a network N with dilation D and minimal dilation E, then $D \cup E$ is a dilation.*

Proof: If $E \subseteq D$, then $D \cup E = D$ is trivially a dilation. Note that D cannot be a proper subset of the E otherwise it would contradict the assumption that E is minimal. Now consider that E is not a subset of D.

As with any set, the cardinality of the union of two sets must take into account not double counting elements found in both sets, i.e.,

$$|T(D \cup E)| = |T(D)| + |T(E)| - |T(D \cap E)|, \tag{5}$$

$$|D \cup E| = |D| + |E| - |D \cap E|. \tag{6}$$

We note that while D and E are dilations and E is not a subset of D, $D \cap E \subset E$ and thus cannot itself be a dilation since E is minimal. From this we have

$$\begin{aligned} |T(D \cap E)| &\geq |D \cap E|, \\ |T(D)| &< |D|, \\ |T(E)| &< |E|. \end{aligned} \tag{7}$$

Combining these facts,

$$\begin{aligned} |T(D\cup E)| &= |T(D)|+|T(E)|-|T(D\cap E)|, && \text{by (5)} \\ &< |D|+|E|-|D\cap E|, && \text{by (7)} \\ &= |D\cup E|. && \text{by (6)} \end{aligned} \tag{8}$$

Thus $|T(D\cup E)| < |D\cup E|$ and $D\cup E$ is a dilation. ■

Lemma 1 *A node that is an in-neighbor of a DCS must point to two or more nodes in the DCS.*

Proof: Let node u have an edge to a node v, which lies in a DCS, $v\in D$. In addition, suppose that it has no other edges terminating in the same DCS. Since v is in a DCS, by definition it must be contained in some minimal dilation $V=\{v=v_1,\cdots,v_m\}\subseteq D$ such that by definition $\Delta_V=1$. Let $U=\{u=u_1,\cdots,u_n\}$ be the set of in-neighbors of V and consider $V'=V\setminus\{v\}$. Note that since v was the only node in V with u as an in-neighbor, the corresponding $U'\subseteq U\setminus\{u\}$ (not necessarily equal because v can have other in-neighbors). Thus we have reduced the number of in-neighbors by at least one, $|U'|\leq|U|-1$, while only reducing the number of nodes by one, $|V'|=|V|-1$. Thus $\Delta_{V'}\geq 1$ so V' is a dilation, meaning V is not a minimal dilation.

Thus u must have more than one out-neighbor in the DCS. ■

Theorem 3 *A node is intermittent if and only if it is an element of a nontrivial dilation choice set and critical if and only if it is an element of a trivial dilation choice set. Otherwise the node is redundant.*

Proof: If a node is an element of a DCS, then it means it is a driver in some (or all) of the minimum control configurations. If the DCS contains only the one node, then this node is always a driver. If the DCS contains more than one node, then the node is in some of the minimum control configurations, hence it is intermittently chosen as a driver.

Now consider a node that is intermittently chosen as a driver. By definition of a DCS, a node not in a DCS is never selected as a driver

First, we consider cycles. If a network has a cycle, it will be used for minimal control since it is self regulating, and thus requires no driver (or rather it can derive control from an existing one). Thus cycles do not contain intermittent nodes, so we assume we're dealing with nodes contained in some stem.

Let n be an intermittent node. Let $C_1,C_2,\ldots,C_k$ be the possible (distinct) minimal control configurations of N (represented as sets of the drivers). Thus for some $i\leq n$, $n\in C_i$, and some $j\leq n$, $n\notin C_j$.

We conclude that n is influenced by one of its incoming edges in C_j, or in other words the cactus that defines C_j has n in a non root position of a stem. Choose the node that acts as its parent node be m. Consider if m had no other out edges: then clearly C_j would not be minimal, since we could continue m's stem with n.

Thus in C_i the stem containing m cannot terminate at m, it has a different child from n, which we call n_1. We hope to show one of m's children is a driver *instead of* n, denoting a choice. If n_1 is a driver in C_i, we're done. Otherwise it is influenced by another node, which directs its stem elsewhere as we observed for n, giving us a new n_2.

We continue this inductively and the finite nature of the network means eventually we must find the node n_r which was controlled directly in C_i. Repeating this construction for each alternate parent node from n in other control configurations, we attain a list of nodes for each parent of n, which we call U. We observe that U is a set of nodes which can be potentially controlled, via their in-edges, by $|U|-1$ many parent nodes (one for every node other than n itself). Thus U is a minimal dilation, so n must be in a DCS, and we're done. ■

Theorem 4 *An edge terminating in a dilation choice set is always intermittent.*

Proof: Note that if a dilation choice set has an incoming edge, it is a non-trivial DCS, since a trivial DCS has only one element and cannot have an in-neighbor. We consider an edge $e=(u,v)$ that terminates in a dilation choice set. By Theorem 3, v is an intermittent node — a driver in some, but not all minimal control configurations.

When v is a driver, then the edge e is not in the matching (or cacti). If e was in the matching and v is a driver, it would contradict the minimality of the minimal control configuration, since v would already be matched (controlled) and would not need to be a driver. Thus e is not in all matchings.

What remains is to show that edge e is in at least one matching. Consider again the scenario when v is a driver and edge e is not in the matching. Because edge e is not used in the matching and v is unmatched, it implies that u is matched along $e'=(u,w)$, where w is a different out-neighbor of u. Consider the new matching $M'=M/\{e'\}\cup\{e\}$ in which edges e and e' are exchanged and v is no longer a driver, but w is. This is a valid maximum matching since $|M|=|M'|$ and one in which edge e is inside the matching, i.e., $e\in M'$. Hence there is a matching which contains e (and corresponding to a minimum control configuration).

Since e is in some, but not all matchings, e is thus intermittent.

■

Theorem 5 *A Type 2 intermittent edge always terminates in a dilation choice set.*

Proof: We consider an edge $e = (u, v)$. If e is intermittent, then it is in some, but not all maximum matchings. When e is contained in a maximum matching ($e \in M$), then in the corresponding control configuration v is not a driver. By Theorem 3, to show that v is in a dilation choice set, it is sufficient to show that v is an intermittent node. Hence to complete the proof, we show that v is a driver in some minimum control configuration.

Let the in-neighbors of v be $\{u, u_1, u_2, \dots\}$. When $e = (u, v) \in M$ is in the matching, without loss of generality, suppose that no outgoing edge from u_1 is in the matching. In this scenario, removing edge e would not invalidate any control configuration, since v could instead be controlled by edge (u_1, v) instead of e. This holds for any of the in-neighbors of v. However, this would contradict the fact that e is Type 2 intermittent, which means that its removal invalidates some control configurations. Thus all in-neighbors of v have matched outgoing edges to other neighbors (Fact 1).

Let the out-neighbors of u be $\{v, v_1, v_2, \dots\}$. When $e = (u, v) \in M$ is in the matching, without loss of generality, suppose that an incoming edge to v_1 is in the matching. In this scenario and knowing Fact 1, removing edge e would require a new control to be added to the unmatched node v. This means e would be a critical edge, and contradicts with the fact that edge e is Type 2 intermittent. Thus, at least one out-neighbor of u must be unmatched in the maximum matching M, say v_1. In the corresponding control configuration, this unmatched node is selected to be a driver.

Note that $M' = M/\{e\} \cup \{(u, v_1)\}$ is another valid maximum matching and would make v no longer matched in M'. Thus in the control configuration corresponding to the matching M', v would be a driver. Since v is a driver in an alternate matching, v is intermittent and, therefore, lies in a dilation choice set (by Theorem 3). ■

References

1. Jia, T. & Barabási, A.-L. Control capacity and a random sampling method in exploring controllability of complex networks. *Sci. reports* **3**, 2354 (2013).
2. Opsahl, T., Agneessens, F. & Skvoretz, J. Node centrality in weighted networks: Generalizing degree and shortest paths. *Soc. Networks* (2010).
3. Watts, D. J. & Strogatz, S. H. Collective dynamics of 'small-world'networks. *Nature* (1998).
4. Milo, R., Itzkovitz, S., Kashtan, N., Levitt, R. & Shen-Orr, S. Superfamilies of evolved and designed networks. *Science* (2004).
5. Norlen, K., Lucas, G. & Gebbie, M. EVA: Extraction, visualization and analysis of the telecommunications and media ownership network. *Proc. Int. Telecommun. Soc. 14th Biennial Conf.* (2002).
6. Shen-Orr, S. S., Milo, R., Mangan, S. & Alon, U. Network motifs in the transcriptional regulation network of Escherichia coli. *Nat. genetics* (2002).
7. Leskovec, J., Kleinberg, J. & Faloutsos, C. Graph evolution: Densification and shrinking diameters. *ACM Transactions on Knowl. Discov. from Data (ACM TKDD)* (2007).
8. Christian, R. R. & Luczkovich, J. J. Organizing and understanding a winter's seagrass foodweb network through effective trophic levels. *Ecol. Model.* (1999).
9. Monaco, M. E. & Ulanowicz, R. E. Comparative ecosystem trophic structure of three US mid-Atlantic estuaries. *Mar. Ecol. Prog. Ser.* (1997).
10. Almunia, J., Basterretxea, G. & Aristegui, J. Benthic-pelagic switching in a coastal subtropical lagoon. *Estuarine* (1999).
11. Baird, D., Luczkovich, J. & Christian, R. R. Assessment of spatial and temporal variability in ecosystem attributes of the St Marks National Wildlife Refuge, Apalachee Bay, Florida. *Estuarine* (1998).
12. Baird, D. & Ulanowicz, R. E. The seasonal dynamics of the Chesapeake Bay ecosystem. *Ecol. Monogr.* (1989).
13. Ripeanu, M. & Foster, I. Mapping the gnutella network: Macroscopic properties of large-scale peer-to-peer systems. *Peer-to-Peer Syst.* (2002).
14. Bakker, R., Wachtler, T. & Diesmann, M. CoCoMac 2.0 and the future of tract-tracing databases. *Front. neuroinformatics* (2012).
15. Burt, R. S. Social contagion and innovation: Cohesion versus structural equivalence. *Am. journal Sociol.* (1987).
16. Adamic, L. A. & Glance, N. The political blogosphere and the 2004 US election: divided they blog. In *Proceedings of the WWW-2005 Workshop on the Weblogging Ecosystem* (2005).
17. Opsahl, T. & Panzarasa, P. Clustering in weighted networks. *Soc. Networks* (2009).

18. Heaberlin, B. & DeDeo, S. The evolution of Wikipedia's norm network. *Futur. Internet* **8**, 14, DOI: 10.3390/fi8020014 (2016).

19. Milo, R. *et al.* Network Motifs: Simple Building Blocks of Complex Networks. *Science* **298**, 824–827 (2002).
